# Supplementary material for: NR2F1 Regulates TGF-β1-Mediated Epithelial-Mesenchymal Transition Affecting Platinum Sensitivity and Immune Response in Ovarian Cancer
Source: Cancers (Basel). 2022 Sep 24;14(19):4639. doi: 10.3390/cancers14194639 (PMC9563458; doi:10.3390/cancers14194639)
Supplement: Supplementary file 1 [file cancers-14-04639-s001.zip › cancers-1887783-Supplementary materials.pdf]

# Supplementary Materials: NR2F1 Regulates TGF-β 1-Mediated Epithelial-Mesenchymal Transition Affecting Platinum Sensitivity and Immune Response in Ovarian Cancer

a

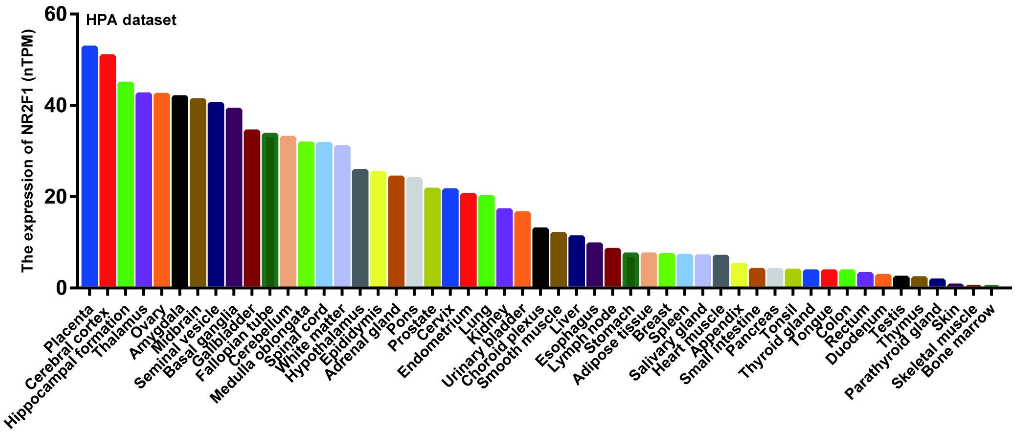


b

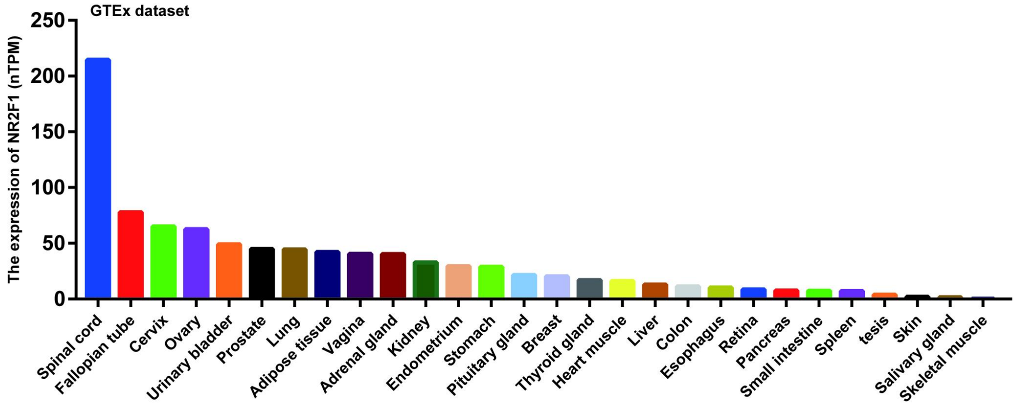


c

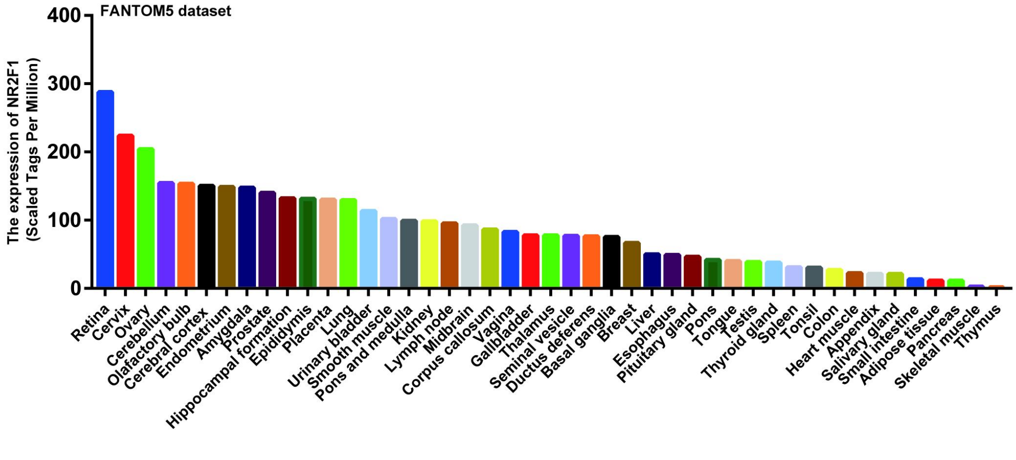


**Figure S1.** Expression of NR2F1 in different normal tissues including ovary tissues using (a) HPA, (b) GTEx, and (c) FANTOM5 datasets.

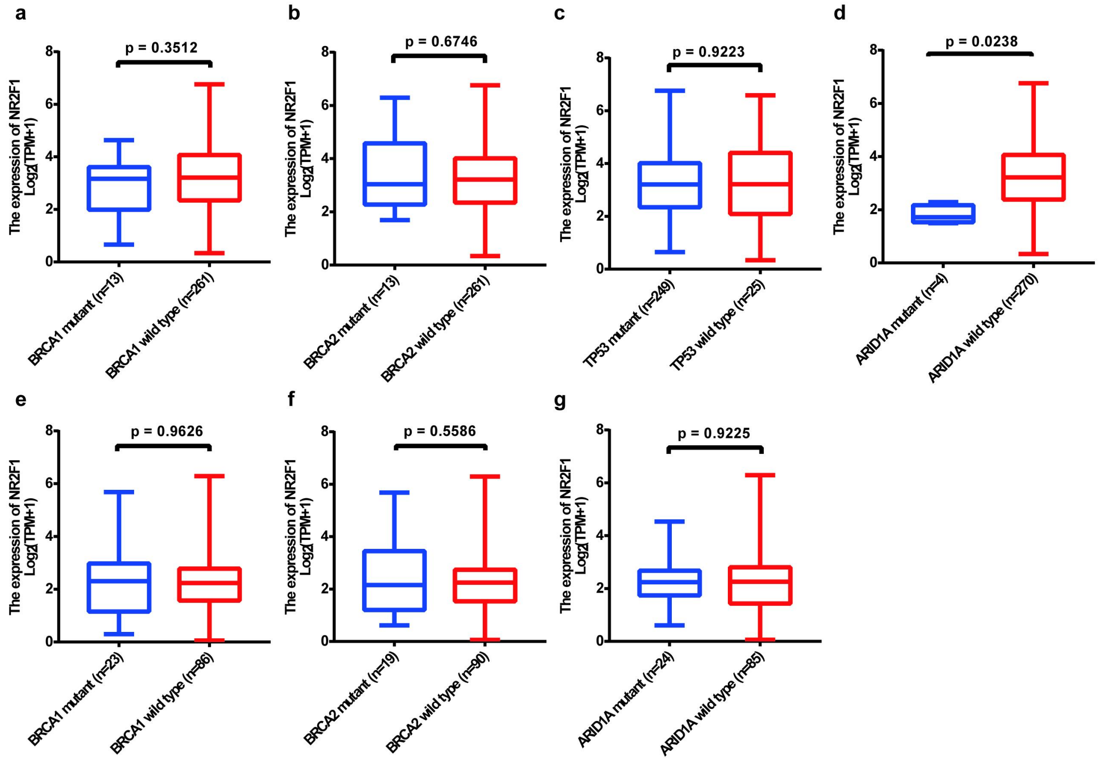


**Figure S2.** The correlation of NR2F1 expression level with specific mutations in OC patients. The correlation between NR2F1 expression and BRCA1 mutation (a), BRCA2 mutation (b), TP53 mutation (c), and ARID1A mutation (d) based on TCGA OC dataset. The correlation between NR2F1 expression and BRCA1 mutation (e), BRCA2 mutation (f), and ARID1A mutation (g) based on ICGC OC dataset.

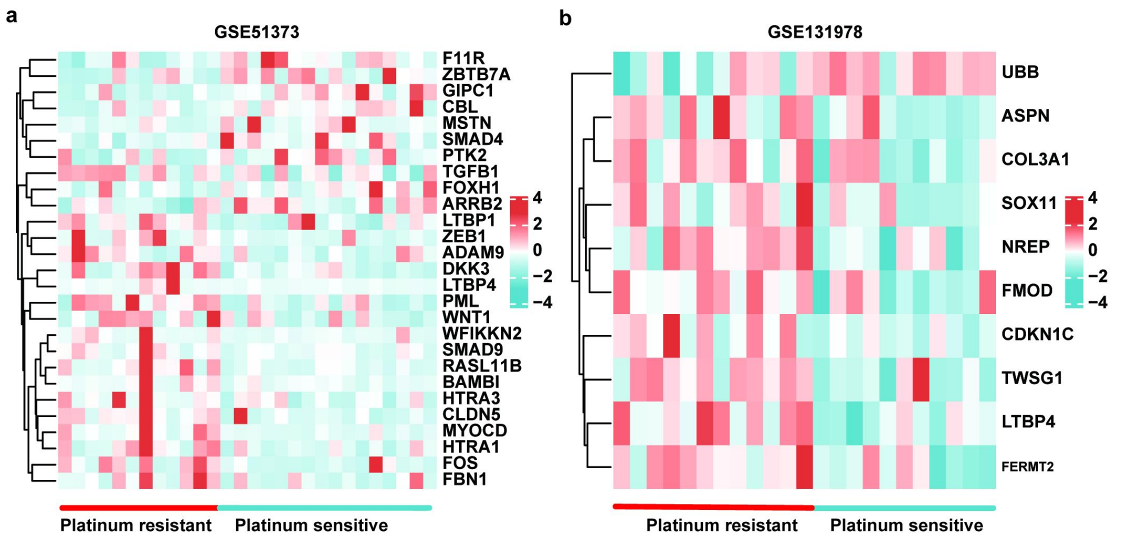


**Figure S3.** Heatmap of the co-DEGs enriched in transforming growth factor-beta receptor signaling pathway in GSE51373 (a) and GSE131978 (b).

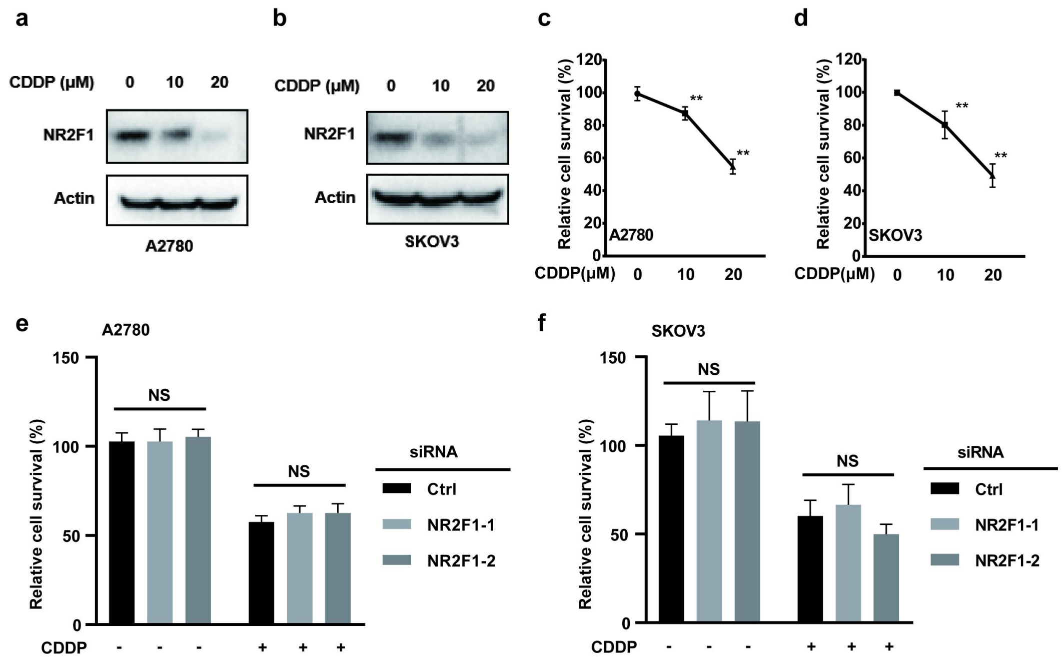


**Figure S4.** Silencing NR2F1 does not affect cell proliferation. A2780 and SKOV3 cells were treated with 0–20 μM cisplatin for 24 h. The protein level of NR2F1 in A2780 cells (a) and SKOV3 cells (b) was examined by western blot. The uncropped blots are shown in File S1. Cell survival of A2780 cells (c) and SKOV3 cells (d) was detected using CCK-8 assay. (e,f) Examination of the viability of siNR2F1-transfected A2780 and SKOV3 cells without or with cisplatin treatment (20 μM) using CCK-8 assay. ** $p < 0.01$. NS: not significant.

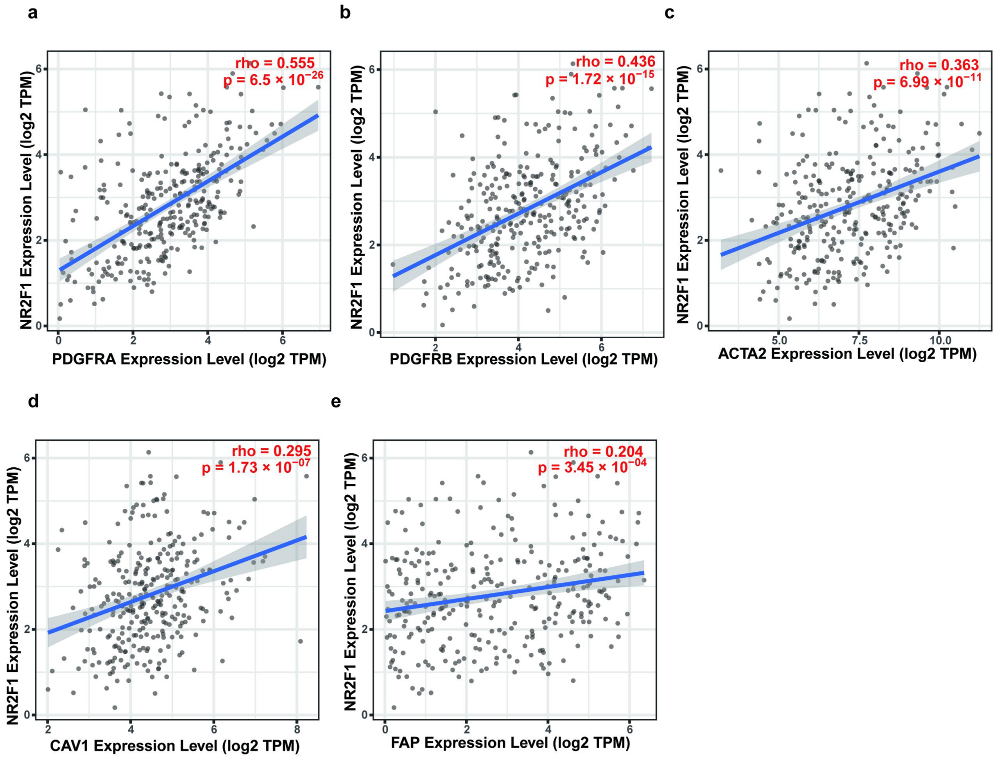


**Figure S5.** The relationship between NR2F1 and CAF markers including PDGFRA (a), PDGFRB (b), ACTA2 (c), CAV1 (d), and FAP (e) according to TIMER2.

**File S1**：uncropped WB images

**The uncropped blots of Figure 5a. (M = Protein standard marker).**

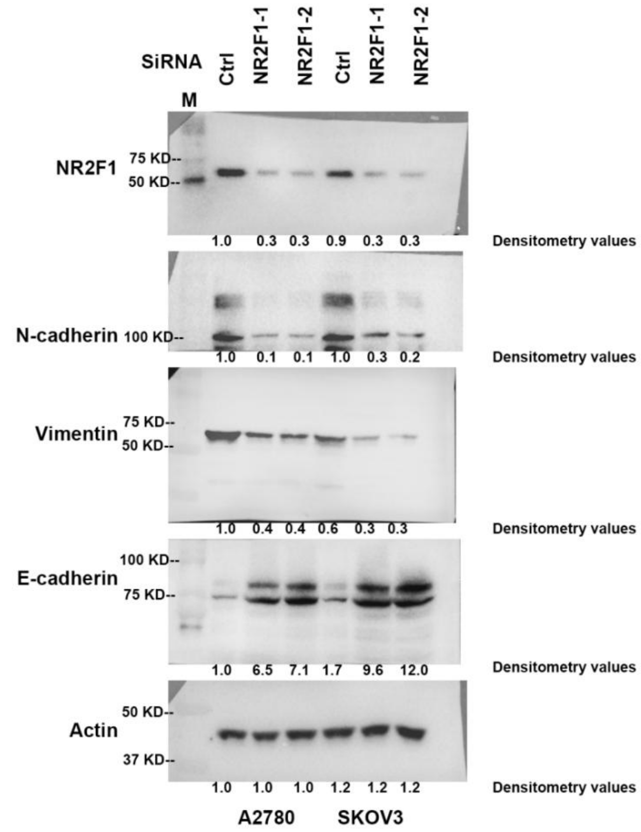

**The uncropped blots of Figure 6b-d. (M = Protein standard marker).**

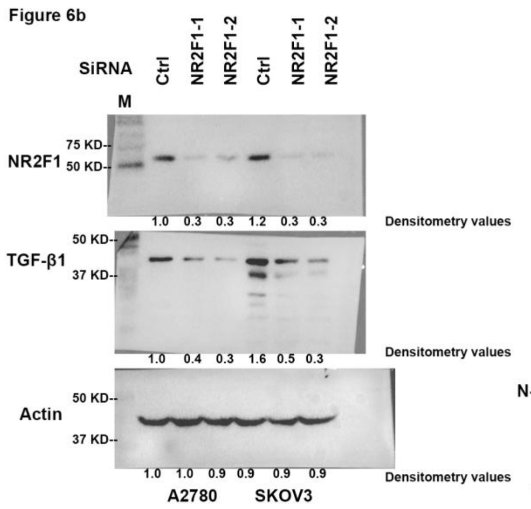


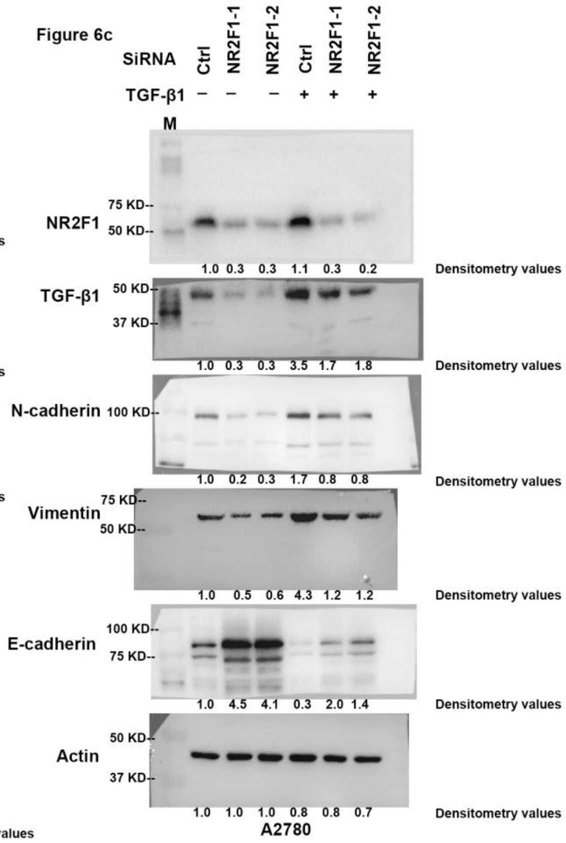


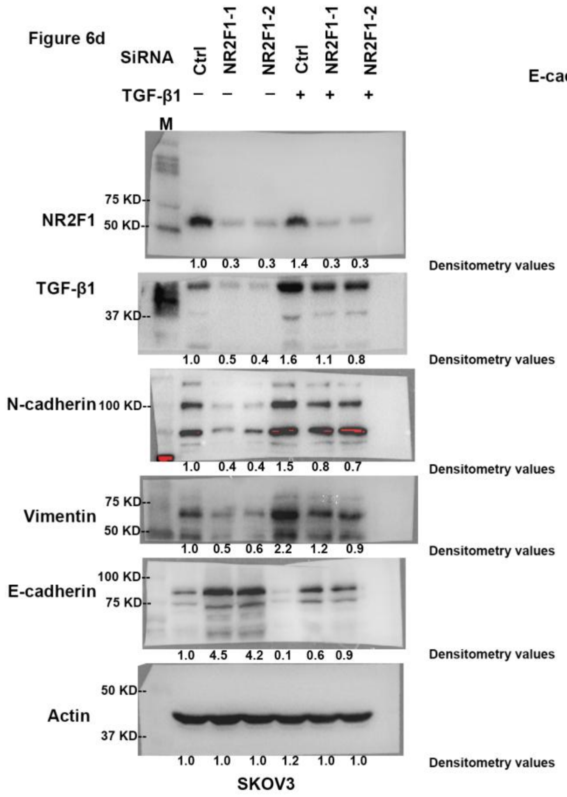

**The uncropped blots of Figure 4Sa and 4b. (M = Protein standard marker).**

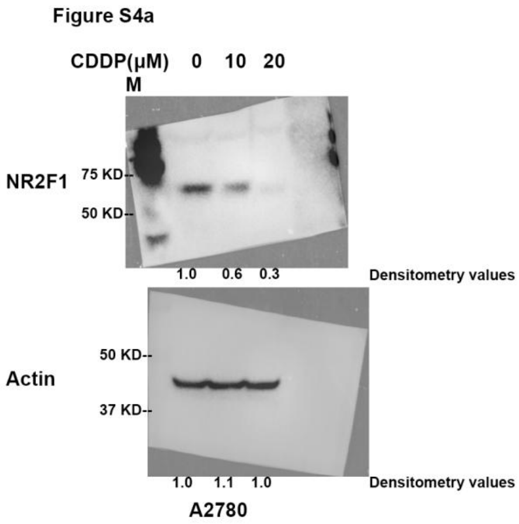


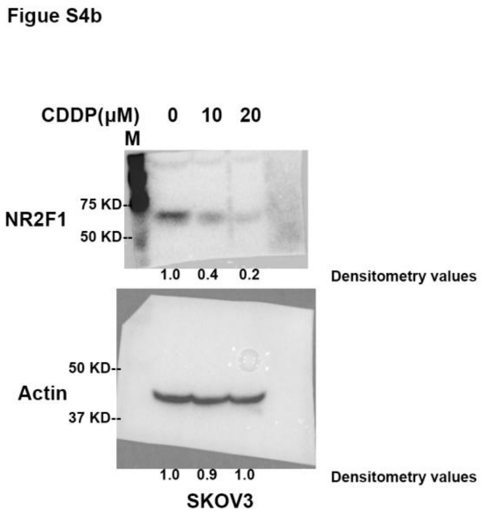

**Table S1:** up-regulated and down-regulated DEGs among GSE51373 and GSE131978

| Datasets | Up-regulated DEGs | Down-regulated DEGs |
| --- | --- | --- |
| GSE51373 | HAND2-AS1 SCARA5 ATRNL1 UBE3A LOC100131655 LRRC75B ADAM22 AKT3 P2RX3 SYNPO2 LINC00908 DENND2A FABP4 GPR156 MAPK10 C11orf96 MGP PDK4 LOC100130872///SPON2 RHOBTB3 CORO2B SCN7A DLG2 RNF213 GNAL OPN4 GH1///CSHL1///CSH2///CSH1 CYP2A6 SDC2 RHOB LOC101928818///PWRN1 EPHA5 C3orf70 ABCB5 GTF2H2B///GTF2H2 VN1R5 P3H2 GEM TMEM204 LINC00673 LINC00161 CPQSNX3 SDHAP3///RGS8 MYT1L FEZ1 DDR2 PDZRN3-AS1 YPEL4 RHOJ BCL2 ARHGAP6 SNAP91 DCHS1 FAM99B SUMF2 PCDH18 LINC01616 MAOB CHGB TNXB TMEM165 IRGQ IGFBP7 ZFPM2 KCNJ8 NBEA ITPR1 PPFIA2 MUM1L1 MRO PML NDNF ZFP36 IGIP GRK5 LOC101805491///TMEM247 ARMCX4 LOC101929174 TEX28 GPC4 ECEL1 CTXN2///SLC24A5 DKKL1 ABCA8 NUDT11 NR2F1-AS1 COL14A1 CCDC83 SMTN MOSPD1 OPN1SW FGF2 CLDN5 SCN4B RYR3 MYH11 DKK3 CAT GATM FKBP7 FGF7 C11orf58 GLT8D2 ZEB1 RASL11B LDB2 GRK4 IL6ST | HIST1H3G TIAM1 PRO1596 C8B PCDH7 PARD6G-AS1 TRIM62 KIF24 MCM8 CTLA4 ITSN1 C1orf74 ZNF417 RIF1 MIR924HG RTKN2 SNX5 GAGE12D///GAGE8///GAGE12F///GAGE2A///GAGE12H///GAGE12E///GAGE12B///GAGE12C///GAGE2D///GAGE12J///GAGE12G///GAGE13///GAGE2B///GAGE2E///GAGE12I///GAGE7///GAGE6///GAGE5///GAGE4///GAGE3///GAGE2C///GAGE1 TEX29 MAVS MYO9B SSX2B///SSX2 SLFN13 GAGE3 H2AFY SLFNL1 ANKRD30BP3 DSC2 OAF THAP2 CCNF SREBF1 SLC22A1 AP1S3 ZXDC MRPL20 HECTD1 CSTL1 HIST1H3F///HIST1H3B///HIST1H3H///HIST1H3J///HIST1H3G///HIST1H3I///HIST1H3E///HIST1H3C///HIST1H3D///HIST1H3A///HIST1H2AD IZUMO2 CNGB1 RBCK1 ARHGEF7 SEMA3F RABEPK PPP1R3E RAD54L CDCA2 NRSN2-AS1 USP2 NDST4 RNF165 SNORD104///SNORA50C ANKS6 ZNF83 EFCAB14 TTF2 PPARGC1B PSMF1 FOXM1 RAB43 HJURP RAB3IP KIF14 SSH2 AFG3L2 UNC45B GTF3C3 PPP4R3B TNRC6C ARHGAP27 LOC339874 SOX12 HIST1H2APS5 IRF4KRAS NCAPH SP100 MIR15A///DLEU2L///DLEU2 LINC00837 LOC100130502 IL2RA AIF1 DIS3L2 C18orf25 ZGRF1 DLEU2 ZNF655 LINC01222 TANK CHST3 TMEM19 GIPC1 DDX11 LINC00667 PRPF38B SMDT1 |

| | | |
|---|---|---|
| | ZNF518BGRIN1 ABI3BP | LOC100506124///TTC21B POLE |
| | ECM2 ANKRD29 WRB | CDK13 USP28 FAM71F1 |
| | FOXP1 WDR72 MEG3 | USP37 LINC01123///LINC01106 |
| | BICC1 JAM3 NSA2 | LANCL1 PRDM14 NUF2 |
| | ZNF717 C1orf21 MYOCD | CYP27B1 SLC20A1 LMO1 |
| | DUSP1 EPS8 FLJ41170 | KIAA1524 DCAF7 ESR1 |
| | MAGEL2 INSR TIMP3 | TCF4-AS1 RSF1 AP1G2 |
| | NR1H4 EGR4 CA4 SPHKAP | ZCCHC3 ZNF417///ZNF587 NR2C2 |
| | LOC100505711 CHML | HIST1H4A GK5 MTF2 |
| | LINC01281 CASC15 | LOC105379143 THRB |
| | CCDC181 ANXA6 AKAP12 | FANCB STK35 REL LOC284080 |
| | PES1 RBM20 WDFY3-AS2 | REXO2 CBX5 SDCBP2-AS1 |
| | LOC100507616///CLLU1 | SGO2 PIP5KL1 KPNA1 FOXN4 |
| | SPATA22 SLC1A6 | APTX SLC26A7 LOC101927204 |
| | LOC339166///WSCD1 SLC18A2 | RAD51AP1 STAT1 LYPD6 |
| | KIAA0825 STX2 | AFF3 RALBP1 LOC283075 |
| | NAALAD2 LAMP2 | FASTKD1 ZBTB40 BROX |
| | CYP2B7P///CYP2B6 CD59 | UBE2O KRTAP7-1 |
| | CHID1 GPRASP1 | FAM72A///FAM72D///FAM72B///FA |
| | PEG3-AS1 NR3C2 | M72C ZBP1 LRRC41 |
| | ZRANB2-AS1 SCN3A NR4A1 | SH3PXD2A-AS1 NAA15 |
| | LOC100287413///GLYATL1 | THADA LOC101928123 |
| | COMP PAX7 NAP1L3 | CCDC88B PATJ UBALD2 |
| | LYPLAL1 LOC285692 | SIGLEC15 IGLL1 PLPPR5 |
| | FAM230B PAMR1 RNF133 | SCRN2 7-Mar PRICKLE2-AS3 |
| | FHL2 PIWIL2 PDE1C | LOC643085 EPG5 OAS3 |
| | CSNK1G1 NUP214 | SLC25A16 PARP9 RIOK3 |
| | ARMCX5-GPRASP2///GPRASP2 | ASPM MIR3916 IL20RB BRIP1 |
| | DACT1 IPO11-LRRC70///IPO11 | ALS2 HSD17B3 SLC30A1 |
| | VKORC1 MFAP4 | PCMTD1 RNF144A-AS1 |
| | LOC100133089 SERPINB3 | KIAA2026 CDC7 ARHGAP39 |
| | TARP TAS2R9 CXCL14 | MAGEA8 BARD1 DTX3L |
| | ZDBF2 SRRM4 NEUROD4 | PLSCR1 ARHGEF39 RBMXL1 |
| | ACTG2 PRTG MAP2K5 | NFYC DUXAP10 AVIL |
| | PRKAR1B LRRN4CL | BCL10 PCGF5 AIFM2 TRMT6 |
| | IGF1 HORMAD2 | ARHGEF12 |
| | SHANK2-AS2 PAPPA | ANKRD23///ANKRD39 DERL1 |
| | SLC1A2 OFCC1 DSTN | AFF4 SOX6 EIF2AK2 |
| | PHYHIP MS4A2 NTRK2 | ZNF606 EPT1 JMJD1C |
| | ANKRD19P FBXO28 GHR | HIST1H2BJ///HIST1H2BG |
| | SFRP1 ADAMTS9-AS2 | DDX39A FLJ32742///DCAF8L2 |
| | ZMAT1 PLA2G2A EPB41L1 | POLQ SSX2IP SFN |
| | ELAVL4 PGR HTR6 FOXP2 | KIR3DL2///KIR3DL1 SMYD2 |
| | EFEMP1 SCN2B | KLHDC7B ACKR4 BTBD9 |

| | | | |
|---|---|---|---|
| | | GH1///CSHL1///CSH1 C7orf50 PLCL1 LOC101928747///RBMX///SNORD61 TEX22 FAM162B PCNX1 ANGPTL1 ARHGAP44 LINC01016 VIM MRGPRF PRDM6 ANTXR2 RBAKDN EGR2 CYBRD1 PTCH2 TEK CARMN///MIR145 ACO1 LHFP MYLK LINC01020 CNRIP1 LOC285097 TRDMT1 HLF CACNB2 C4orf46///TOMM7 CACNG2 KIT EEF1A1 FOXE1 CLN8 GAB1 LOC101929713 FAM126A MAN1C1 SLC22A7 C8orf49 MMGT1 PDE7B VIPR2 CTD-3080P12.3 ZCCHC12 TMEM106B RPS4Y1 C4orf3 COL4A6 HAS3 MSRB3 LAMA2 HEXB HHLA2 FOS FOXL2 TFPI RNH1 SPTBN4 TRPM3 NXPH3 PDE2A STARD13 GNG11 AGFG2 DNM3OS EXTL1 CLMP AOX1 RNF144A HMGCLL1 PLS3 STAT5B SMIM20 MAOA PQLC3 LMOD1 THSD7A VWF RGS5 PDGFD PDE5A C5AR2 CCDC127 NT5DC4 PTGER3 GABRB2 PRPF4B FBXO32 ZNF582-AS1 ADH1B IGFBP6 PDLIM3 LINC01583 CD79B ZDHHC4 CLSTN2 MBNL2 NR2F1 OR7C2 MTTP PLNTIAL1 PLPP1 FAS CYP2B7P LOC102546299 LOC101928307 DKFZP434L187 PROX1 FAM46D EIF1 SLC35B4 NID1 USP46 PTHLH ACADL PRKD1 CTGF TLX3 WISP2 UBE2W PSORS1C3 | CD2AP UBA6 PRKD3 MAGEA4 LINC00305 SHISA8 FANCI DDX51 ZNF484 HSH2D KRT81 ERICH4 MIR6125///USP15 GAGE12D///GAGE8///GAGE12F///GAGE2A///GAGE12H///GAGE12E///GAGE12B///GAGE12C///GAGE2D///GAGE12J///GAGE12G///GAGE13///GAGE2E///GAGE12I///GAGE7///GAGE6///GAGE5///GAGE4///GAGE2C///GAGE1 DNA2 ZNF460 QTRT2 FNDC3A MIR6758///MARS DYNLL2 NEK2 IGK///IGKC OSBPL3 H1F0 MIER2 MYL12A XRN1 TP63 NAPG LRRC37A5P ZNF587 QSOX2 LINC00459 SNAPC3 ESPL1 CES3 ISY1-RAB43///RAB43 IDH3B LOC101929517 LOC100420758 ATP2B4 SMR3A///SMR3B PER3 DCBLD2 KCNB1 RS1 SAMSN1 CDC20B IFI44 PSRC1 CD84 TBX3 FAM111A C3orf52 ESPNP///ESPN ZNF367 CSRNP3 SLC30A6 F11R TAF5 PKN1 LSM14B MIS18BP1 PLCH1-AS1 ZNF551 IQGAP3 LOC101928551 LHX4-AS1 PHF19 NDC80 CCDC150 FANCD2 LOC105376166 LOC285500 LOC100507377 GPSM2 GGT6 LINC00470 SYNJ1 AFF2 CYP19A1 DCAF8 RNF217-AS1 LMNB1 EWSR1 POLL CCDC18 LPIN1 BEST3 CDC42BPG CCDC30///PPCS GPR157 SEMA6A LOC146795 INTS6 LMF1 DENND2C PEX26 CBL CKAP2 NDUFS1 RGS14 BCL2L11 MRPS12 OVOL2 MAP4K2 ODF2L HAUS8 BCL11A CLIP1-AS1 HINT1 |

| | | |
|---|---|---|
| | MCHR1 LAMA1 PF4V1<br>CNIH3 DMRTC1B///DMRTC1<br>FIBIN LRRC17 PDE8B<br>ARRB1 FAM201A SNHG24<br>GDF7 PDZRN3 MBTPS2<br>SCRT1 IGFBP7-AS1<br>GNPDA2 DNAH17-AS1<br>FMO2 WFIKKN2 CUBN<br>ESD NR4A3 RBPMS LIX1L<br>NR2F2-AS1 LOC286114<br>RASD1 PPP1R1A HACD1<br>PRSS58 BOD1L2 DSCAM<br>EMCN C2CD4C TPSB2<br>HELQ RTTN ACSL4<br>DIO3OS PLD1 TRA2B<br>DMGDH PDPN TEAD2<br>TCF21 NRXN2 LINC01225<br>CYGB ANO4 AP2A2<br>FLVCR2 ZNF385B<br>LOC101928847 ANKRD10<br>KIAA1652 GAMT<br>LOC100506731 C3orf14<br>NEGR1 MIR5188 SUN5<br>RYR2 ADCY2 CCL21<br>RPS15A TST MRPL20 EIF1AY<br>MAP7D3 FGF7P3 ATG5<br>KDELR2 LINC00163<br>LEFTY1///LEFTY2 VGLL3 CA6<br>M1AP TCEAL7<br>LOC102724094 ATP11C<br>LINC01279 KLK15<br>MAGI2-AS3 LOC105371307<br>ARHGEF28 TAS2R1 CXorf57<br>STAMBPL1 FOXG1 NDN<br>RPE65 ADH5 CYP27C1<br>GNE BMP5 GRM5<br>LOC100507403 FHL1<br>CRCT1 CSHL1 PSMG4<br>PRR16 ZNF653 MATN3<br>RASA1 RNF148 TIGD4<br>FCGBP POLR3G FAM13C<br>LARGE1 GPR78///CPZ CEP85L<br>P2RY1 COX7A1 GULP1<br>ABCA9 FAXDC2 APOD | MGC12916 BOC HOXB-AS3<br>RBM25 ETV6 NRSN2<br>LRRFIP1 TFB1M DDX42 MDM1<br>LOC283485 CLIP1 PDE4A<br>SP110 TMEM225 LYPD6B<br>UTYDDX50 TACR3<br>TP53TG3HP NUSAP1 RPS10P7<br>POLR3G ESRRB RTFDC1 KIF2C<br>PPHLN1 FAM205BP PCP2<br>GJB3 CDH19 ZBTB7A<br>SCGB1D1 LOC151657 LDB1<br>FANCA MYH8 H2AFY2 CDH6<br>SNX29 IRF5ESCO1 MCOLN2<br>HS6ST3 SLC6A19LGALS13<br>ATP2B3 CNOT2 TRPC3 EML4<br>LOC100129175 CSNK1A1<br>CEP76 TNRC6C-AS1 PGP<br>LOC102725022 USP32///USP6<br>SNRPD1 U2SURP FAM174B<br>SOCS1 CDC25B ZNF682<br>KBTBD12 DEFB126 SLC35B3<br>CCL13 RCBTB1 SRMS PAQR4<br>KIF26B SEC14L2 MYCL UTS2<br>SMAD4 SHMT1<br>GAGE12D///GAGE12F///GAGE12H<br>///GAGE12E///GAGE12B///GAGE12C///<br>GAGE2D///GAGE12J///GAGE12G///GA<br>GE13///GAGE12I///GAGE7///GAGE6///G<br>AGE5///GAGE4///GAGE1 ZSCAN30<br>LOC101929774 EPB41L4B<br>GALK1 LOC100996263 HID1<br>MIR7703///PSME2 CCNE2 LMO4<br>CCL28 FOXJ3 PEX13<br>TROAP CDT1 PP12719<br>LRRIQ1 E2F7 CLSPN<br>ALDH3B2 CABP5 TOP2A<br>AMPD1 KDM8 AP1AR GEN1<br>GPR19 HIST1H3D///HIST1H2AD<br>CTTN NKX3-1 HIST1H2BM<br>NIPAL3 CENPF PPP5C<br>ZNF818PKCTD5 MACROD2<br>SLCO4C1 GTF2IP7 LOC401324<br>ETV1 CENPL HRH4<br>DENND1B LINC00202-2 RBM33 |

| | | |
|---|---|---|
| | LINC01103 LOC100996506 LOC150005 ZCCHC18 CLIP2 BHMT2 DGCR5 SEL1L2 CACTIN MBLAC2 FRMD4A LOC100422737 SLC6A17 AKR1C3 ITGA1 NR2F2///NR2F1 TSPAN5 PLEKHG5 LPAR4 POU5F2 HECW1 IQCF2 LOC339298 TBC1D8B DMD SOD3 LOC101927044 GRIK2 RNF8 TNFSF18 SNTG2 ICOSLG RNF32 MYLIP ITM2A NOCT DSCC1 BAMBI SLITRK4 SNAP25 MAST4 HGSNAT SNX32 YES1 LOC283484 ADAM23 CASD1 CYTH4 HTRA1 SHC4 ADAM11 ACSS1 STXBP5 LINC00593 F5 MAP4 CDIPT-AS1 CAPN6 EPHA10 FBN1 SH3D19 ARL11 HBB CENPI ACKR1 UGT2A2///UGT2A1 PMP22 FOSB LOC101928284 HBG2///HBG1 TINCR GABRA2 CMBL KIAA1462 TRIM23 USP53 MIR4755 PCDHA2 ANGPTL2 BFSP1 C6orf48 HOXA-AS2 SERINC2 RPGRIP1L INF2 RAC1 KDSR GALNT9 NDUFS4 CC2D2A FBXL7 RBMS3 PNMA2 EGR1 PCOLCE2 KMT2D CECR2 CST9L CD22 SH2D6 BSG MAMDC2 SLC22A3 MFAP5 FGF13 CNN1 CYP2U1 TCEAL1 MIR3663HG SGCE NELL1 ELOVL4 LOC100506718///FLRT2 NOS1 LOC200830 ASPG LOC101927534 BMP2 | MAGEA6///MAGEA3 P2RY10 FXR2 BUB1B PMCHL1 ALCAM DACT3-AS1 KLC4 PHF20L1 NAT16 GABRG3 MCM10 MEIS1 MIR205HG PKMYT1 IL22 RPRD1A SLC19A1 FMN1 ST8SIA5 USP48 LOC100505666 LOC100289518 NTRK3 SQSTM1 LOC283856 CASP8AP2 LOC101929819///LOC100133331///LOC100133182///LINC01001///LOC100132062///LOC729737///LINC01000///LINC01002 METTL7B CCP110 KIF18B TREML3P ZBED6 GHRLOS FBF1 C11orf88 PARP14 ALDOB LOC149684///BPI LOC101929368///FKBP1A LOC102725382 EFNA2 SYNPO OFCC1 ATXN1 ZNF814 PRR11 ZNF93 FAM110A KDM4C TUB FOXH1 TNFRSF13B NUP160 HCP5B PTK2 TFG LINC01398 NADSYN1 DNAH14 C2orf54 GUSBP1///GUSBP4 PVR CDKL3 DCLK2 METTL10 SLC35F6///CENPA LOC101927526 MAPK6 MYRF EME2 IL23A RRP7BP///POLDIP3 ORMDL1 HGSNAT MIR1178///CIT POLR2B NPM2 TRIM11 HIST1H2BD RAB30 CEPT1 MCMDC2 TTC7A ZNF24 ABCA5 SP140L LMBRD2 RRP8 VIPR1 THUMPD3-AS1 FBXO10 EPHX4 LOC101928269 IFIT2 ZNF587B TUBA1B ASXL2 ALDH1A3 GTSE1 GJC1 ZDHHC17 MKI67 PTBP3 OR6B1 LOC51145 STIL ZFP41 C6orf62 FZD5 TCF15 THRIL///BRI3BP RGMB-AS1 ZNF215 TCL6 MEIOC SCYL2 |

| | | |
|---|---|---|
| | CD160 C15orf59 HTRA3 CFAP46 LOC101928460 PLA2G5 ENPP6 SCTR NCAM1 CD6 ZSWIM5 ELN PMP2 TUG1 PALLD CACNA1H DIRAS3 GALNT15 HSDL2 GFRA4 TTYH2 CALB2 MAP6 MLXIP OSBPL6 ARHGEF10 CDH11 LOC101930322///ABCC6P2///ABCC6P1///ABCC6 CACNA1C THBD C5orf47 KANK2 KCNE4 WASF3 LTBP1 L3MBTL3 MSMB CECR5-AS1 FBXO36 WHAMMP2///WHAMMP3 DUSP26 MAT1A SIGLECL1 MYLK3 SYTL4 ZP4 KIAA1549L UST-AS1 RFX2 RGS2 NRXN1 ANK2 ST8SIA2 MMP28 MSRA F2 WFDC6 MEDAG WDR86-AS1 SLC25A31 CXCL2 CCDC178 LOC101927798 TRIM50 FAM49A CCDC80 SEMA6A PCDHA2///PCDHA3 STXBP5L TMEM161B-AS1 KLHDC1 GLB1L3 CTD-2194D22.4 AKR1C6P PARVB TMCO5B ACTL7B TPSAB1 TCEAL2 GEMIN8 ABCA6 CEP44 BIN1 GLRA1 C4orf17 GPR20 HSPA2 NRAP HS3ST1 ASPH LOC441052 PRSS35 SEC31B MTUS2 KCNA1 TRIM61 MMP16 ARHGEF26 TDRD10 NIPAL4 KLHL35 RNASE7 SOBP KSR1 MYCT1 LTBP4 LOC101930243///LOC101929865 EGR3 ERF LEPROT///LEPR LHX9 SPARCL1 SLC9B2 | TTTY13 FOXK2 PIAS2 NAB1 NOP14-AS1 CIITA CERS6 STX16 GPR180 NFIX RIBC2 IFIT5 KCNK9 LOC389834 DENND2D GPD2 FCER2 GIGYF2 MTHFD2L SVOP FAM81A PLAC9 CTAGE8///CTAGE4///CTAGE9 CNOT7 WDFY2 RDM1 UNC5C ANAPC5FABP6 CELF2-AS1 C18orf21 ATF7IP2 ANPEP C14orf159 SKAP1 MDM2 ADAD1 LINC01127 CKAP2L KLK14 LINC01410 EPN1 WNT7B LOC101927623 CTAGE8///CTAGE4///CTAGE9///CTAGE15 CAND1 PLEKHM1 SSX3 SGK494 SDK2 ANKRD12 LOC100289283 EPHA4 RUNX3 CENPM CD209 HMGA2 TRIM36 OR1G1 PLEKHA8 LOC101928907 HIST1H1T LOC643355 ZNF75A STRBP ROPN1 TRUB1 LINC01346 NMRK2 RECQL4 LOXL4 KCNT1 CCNO MGAT3 PRDM10 ZNF295-AS1 DNASE1 KLHL28 GABRB3 PRR34 SLAMF7 FBXO3 ZWINT CFLAR RCOR3 MIR6773///ESRP2 BIRC5 UBE2G2 IFIH1 PHF6 ECT2 CLEC16A TIA1 PNMA3 LOC101927702 ETV4 NPAS3 C9orf47 LOC101928708 NUP210 HMMR ST6GALNAC4 KCNAB3 ARMCX4 PPP1R12B HELQ RAP1A HELB GRK3 ARHGAP26 BRCA2 LOC101928623///LOC401320 M1AP GTPBP3 ZNF789 RFX7 AGFG2 LOC102800447///LOC101930566 RBBP9 MARVELD3 GTPBP10 SMIM14 LOC338694 WNK1 |

| | | |
|---|---|---|
| | FLJ22763 TGFB1 HNRNPDL RNF180 ATE1 LOC100506286 GABARAPL3///GABARAPL1 RFX4 RUNDC3B NBPF20 FBXW12 KLHDC8A SLMAP CAPNS2 GART LINC00608 CADPS2 CD36 UNC80 CD300LG SGIP1 FAM114A1 GGTLC2 OGN PTPRB JAM2 KIAA1210 WWC2 SLC6A1 SLC43A1 TMCC1 RORC TAF4 IL13RA2 EML5 SHE ARG1 FAM43B C7orf77 FOXO1 CEP128 TXNL1 TRPC5 OR7A17 FLJ30901///SCUBE1 MRVI1 ADAM9 GLUD2 MPL PRELP RNASE4 ANOS1 PLCXD3 MALSU1 RECK TAGLN RAP1GDS1 ERVH-6 RPS23 SIM1 MATK XRCC4 TSPY1 C14orf105 LCMT1-AS2 VSTM2A-OT1///VSTM2A LOC100294145 PRSS53 SIK1FILIP1L SMAD9 HMMR-AS1 PRMT1 POLK LRAT ADCY8 FNDC4 NAV2-AS4 CLVS2 ENPP3 SGPP1 ANTXR1 B3GALT6 COL2A1 ARHGAP31 CSF2RA CSGALNACT1 LHFPL3-AS1 ACTA2 PCP4 ARHGAP24 SLC16A7 ABCA13 BRSK2 CLEC2L TMED4 LOC101927331 ENTHD1 DPYSL2 WNT1 ZNF396 DLGAP1-AS3 PPP5D1 TMEM200A TEX13B SCN1A SETD7 CASKIN1 VNN3 SPIDR ATP1A1 SCG5 CCL15-CCL14///CCL14 HPCA LOC100505978 MBNL1-AS1 | LOC101926942 SFT2D3 STX19 SLC45A2 MLPH TACC3 LOC101927690 RASA3 LOC286359 PGAP1 PPARD SIN3B GPR158 DCAF15 ANKRD44 LRRC37A3 TMPO-AS1 PCDHB1 DOCK4 MYT1 GNB1L ARHGAP35 PLGLB1///PLGLB2 ZKSCAN7 ASB16 COL8A1 STAG1 LINC00491 LCA5L NUP93 IFIT1 APOF PIF1 SAMD9 BAZ1A ANKRD65 FAM64A DNAJC9 COL9A1 LMOD3 EPRS CADM3///ACKR1 GAS2L3 TMEM161B CEP152 TPM4 C21orf58 GINS1 CARD18 PIK3R3 SOS1 LOC729224 ZNF221 STK4-AS1 CITED4 SSU72 WDR76 ZNF519 GEMIN7 ARTN CLIP4 HMGA1 SH3TC2 ERV3-1 NLGN4Y MSTN NECTIN4 NNT KIF23 DGCR7 IFNL1 NAA40 ACKR2 TMEM110-MUSTN1///TMEM110 ZNF107 DIAPH3 ALG10 RBM15 CSH1 SERP2 FCRL5 HOXD13 FAM133DP///FAM133B CERS5 ATL2 FAM65A TSNARE1 MAGEA1 CD28 SCIMP ZNF326 LTK NFIB DR1 ARPIN ARRB2 LITAF SERPINB5 LOC101930531///LOC101930288 SRSF11 SPTAN1 LOC100996906///CDRT1///FBXW10 MIR4290HG FOXJ1 ISG15 MASTL PLAC8L1 CEP55 DCP2 LOC389641 PPIL2 RAD52 BTBD11 RTP3 LRRC37A4P ARPP21 ADGRF3 SP140 TYMS LOC101930112///SPG7 TMTC2 GTF2A1 LOC101927348 |

| | | |
|---|---|---|
| | RALGAPA1///RALGAPA1P1 PYGO1 SORCS1 IGFBP4 PPID BAG2 PDGFRA LRFN3 INMT TMEM47 GBP6 PAQR9 TACC1 OLFML2A GZMM MMP20 MIR6883///PER1 NEFM RBP7 TTC28-AS1 LINC00967 SMOC2 MRAP GABRA6 IFNA1 LOC100506563 KCND2 MAGEB3 SLIT3 PTPN5 NKAPL LMO3 CCDC42 LY75-CD302///CD302///LY75 CHST7 PRRT2 C4BPA PLEKHG2 GLI3 HSD17B2 LIMS1 IL33 MDM2 GFPT2 REPIN1 TXNIP DPY19L2 TTC22 LOC101928909 ZNF207 KCNK1 KBTBD11 CEBPD BMP6 ZCCHC14 KCNC1 TRIM24 PLPP7 KCTD17 PMS2P4 SELM ARSD LINC01140 LOC105376944 RAPGEF1 FBXL13 MOGAT1 KL LRFN5 GOLM1 TLL1 PIK3IP1 MMP8 ANKRD34A FBXO17 PRKG1 KIDINS220 DFNA5 TIMP2 SLC2A13 ANGPTL7 LOC105370580 ST3GAL4-AS1 FAM189A2 LOC105377924 WAS ADGRA2 TCEAL3 GBA3 PCSK9 C1QTNF4 C2orf80 FGF22 ERC1 TMEM178B ACACB DNAJB8-AS1 ISL2 PSMB8-AS1 TMEM74 ADAM3B NFASC LINC01187 KCTD16 LOC102725315///LOC727983///POM121L10P///POM121L4P///POM121L8P///POM121L9P///POM121L1P | MYO1H PTPN14 WDHD1 ELF3 PLCXD1 ZNF334 TXNL4A BRMS1L CARF LARP6 FBLN1 CMTM7 KIAA1804 MAP2K7 PAPPA2 PLPP2 LRMP ATAD2 |

| | | |
|---|---|---|
| | PCSK1N TRPM6 HILS1<br>PSAP PRB4 EDNRA<br>FRG1CP///LOC283788 PIGZ<br>MMP24 TBX3<br>LOC100507311 FMO5<br>MYL4 TDH PDGFRB<br>PCDH15 NMUR2 SLC30A4<br>KLHL13 NPY2R NUDT10<br>COPG2 KLF6 NCR2<br>OR9A1P SMIM4 DDIT4L<br>BCL2L15 ZNF44 GABARAPL1<br>PLXNB3 TMEM185A<br>TMEM187 GPIHBP1<br>DRD3 CD99<br>GALNT8///KCNA6 DCN<br>TMEM207 LSAMP ABHD1<br>PRPF18 CTSF<br>CNTNAP3B///CNTNAP3P2///C<br>NTNAP3TTTY5 EGFR IGFBP1<br>HAND2 TSPY26P NAP1L1<br>SEMA5A PROK1 OPN3<br>CA11 PTRF ERICH1<br>CARD8 MCF2 CLDN11<br>DET1 CADM3 NAPA-AS1<br>PTOV1-AS1 COL4A4 GJD3<br>MEX3B LINC01115<br>ZFYVE28 UMAD1<br>LINC01089 CPE GNG7<br>LDLRAD4-AS1 SNCA<br>CEP57L1 TFRC ATP6V1C1<br>MAP1B | |
| GSE13197<br>8 | ESF1LOC101928830///LTA4H<br>ARL4C NAP1L1 SMARCA1<br>SOCS5 TCF4 PSMF1<br>EMX2 LTBP4 OSBPL8<br>CNOT2 RNFT2 C12orf29<br>ACAA2 HDAC4 ACKR3<br>ZMYND8 CBX1 NREP<br>IL11RA CSNK2A1 NELL2<br>MIR1292///SNORD110///SNORD<br>86///SNORD57///NOP56 DTYMK<br>DESI2 NR2F1 HJURP<br>MYH10 DIP2C URI1 | HIST1H2BD GSDMD CLDN10<br>GBP2 GPAA1 TAPBP<br>HIST1H2BE GRINA<br>HIST1H2BC///HIST1H2BI///HIST1<br>H2BE///HIST1H2BF///HIST1H2BG<br>PYCRL OR7E14P<br>MIR6834///PFDN6 BTN3A3<br>ASRGL1 HLA-E SLC52A2<br>PRUNE2 SNX19 HOXB6 TAP1<br>HLA-G SPCS2 TSTA3 GBP1<br>PRSS16 GSN HIST1H2BH<br>YIF1A FAT2 HSDL2 UBB |

| | | |
|---|---|---|
| | SUGCT MIR15A///DLEU2L///DLEU2 DYRK2 PLEKHF1 EIF4E2 LSM8 MEST EFNB2 AGAP1 TM7SF3 VCAN FERMT2 TUBB6 FXYD5 MFAP2 TROTNKS CENPF TPPP3 SLC16A1 LSM14A PCOLCE UQCRFS1 CCNE1 IGFBP6 NUAK1 PALLD PPP1R12A ZIC1 ROBO1 NUP107 GPM6B HMGB2 ZNF292 FKBP1B TWSG1 IGFBP3 ATP1B1 EFNB3 CDKN1C PON3 MIR4738///H3F3B///H3F3A CPVL FMOD TIMP3 ASPN TPGS2 PLCB1 MXRA5 POP4 SNCA PEPD F2RL1 PRSS2 ISLR RGS4 DIEXF IGF1 LOXL1 NDUFA4L2 COL3A1 SPRED2 PTH2R CDH18 SH3BP4 SLC38A1 EMP1 MYO1B TMEM45A BGN CDH11 TUBA1A RHOBTB3 SMC3 SOX11 COL11A1 ACTA2 SSPN COPS8 SPARC UBA2 SRPX PXDN COL5A2 TNFAIP6 IQCJ-SCHIP1///SCHIP1 ZNF124 FABP4 HSPA2 PLOD2 MSANTD3-TMEFF1///TMEFF1 HOXC6 ALDH1A3 TTK | VPS13C DGAT1 IDH2 UBBP1 LY6E HLA-C TRIM27 H2BFS DEPTOR LAMP2 ENDOD1 CFB TAP2 HIST1H2AC KRT23 TTC9 PSMB8 EXOSC4 FBXO9 FZD4 MPZL2 YME1L1 LACTB2 LOC101060835///HLA-DQB1 HIST1H4H RIDA HIST1H1C PSMB9 FNBP1 APOL1 FZD6 CXCL11 ANO1 HLA-DPA1 ST6GAL1 PPP2R1B HLA-F C8orf33 CYC1 PLEC CXCL10 HLA-B CYP11A1 SERPINB1 SH3GLB2 CRISP3 IDO1 HIST1H2BK DNPH1 SORL1 TFAP2C HLA-DQB1 ACSM3 MIR7112///BOP1 ACTG1P4///AMY2B///AMY2A///AMY1C///AMY1B///AMY1A TPK1 RARRES3 SH3GL3 LIFR HIST2H2AA4///HIST2H2AA3 WARS HOMER2 VGLL1 MIR452///MIR224///GABRE CD74 CYP3A5 RBPMS HIST1H2AE AQP5 VAV3 OPLAH CXCL9 SLC7A11 PALMD CA2 HLA-DMA LOC100509457///HLA-DQA2///HLA-DQA1 MIR7113///MIR4691///NDUFS8 HLA-DMB LOC100996809///HLA-DRB4///HLA-DRB1 TACC1 GREB1 EGLN3 XIST HLA-DPB1 |
